# Supplementary figures and images for: Effects of deuterium oxide on cell growth and vesicle speed in RBL-2H3 cells
Source: PeerJ. 2014 Sep 2;2:e553. doi: 10.7717/peerj.553 (PMC4157235; doi:10.7717/peerj.553)

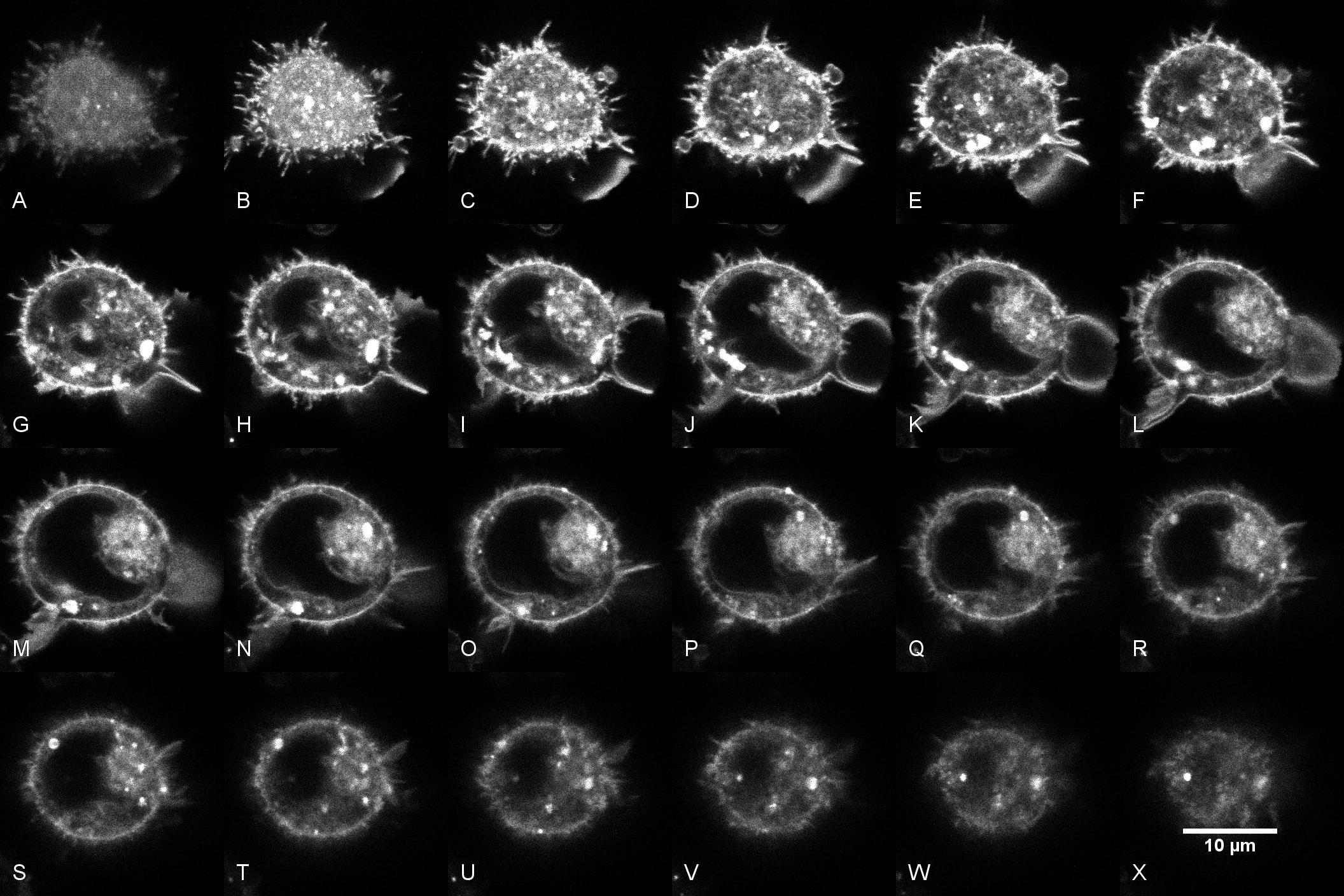

Supplement: Figure S1 — To confirm incorporation of diI into the cell plasma membrane and membrane-bound organelles, diI-stained RBL-2H3 cells were imaged using a confocal microscope. Cell samples were maintained at 37 °C using an objective heater. For confocal imaging, a Leica TCS SP5 confocal laser scanning microscope with a 63 × oil immersion objective was used. diI-labeled cells were excited with 543 nm laser light in confocal microscopy. Appropriate filter settings were used to collect fluorescence. Figure S1 depicts a confocal z-stack montage of a diI-labeled RBL-2H3 cell and shows that diI labels cell membrane barriers, including the cell plasma membrane and membrane-bound organelles such as the nucleus, lysosomes, and endosomes. This figure shows a confocal z-stack montage of a diI-labeled RBL-2H3. All panels correspond to individual confocal z-slices starting with the z-slice of the cell in contact with the glass substrate, panel A, and going up to the top of the cell, panel X. The z-step size was set to 692 nm. Scale bar represents 10 µm. [file peerj-02-553-s002.png]
