# Supplementary material for: Effects of deuterium oxide on cell growth and vesicle speed in RBL-2H3 cells
Source: PeerJ. 2014 Sep 2;2:e553. doi: 10.7717/peerj.553 (PMC4157235; doi:10.7717/peerj.553)
Supplement: Supplemental Information 4 — LMD files are the Flow Cytometry raw data for Fig. 2.PDF files are overview pages showing analyzed data with the applied gatesFile name description:dayoneH2O: one-day old culture with 0 mol/L deuterium oxide dayfourH2O: four-day old culture with 0 mol/L deuterium oxide dayoneD2O: one-day old culture with 15 mol/L deuterium oxide dayfourH2O: four-day old culture with 15 mol/L deuterium oxide. [file peerj-02-553-s004.zip › Flow Cytometry Data/day_four_D2O.pdf]

Playback Institution:

Protocol :rblcountpianal.PRO

New Protocol

Analysis Date: 20-Nov-2013, 16:40:31

Settings File: rlbcountpifix.PRO, 20-Nov-2013, 16:31:53

Listmode File: 10_WJT 00012651 2013-11-20.LMD

Run Date: 20-Nov-13, 16:32:28

Sample ID: Well 10

User ID: WJT

Acquisition Time/Events: 100.6s / 6079

[Ungated] SS Lin/FS Lin - ADC

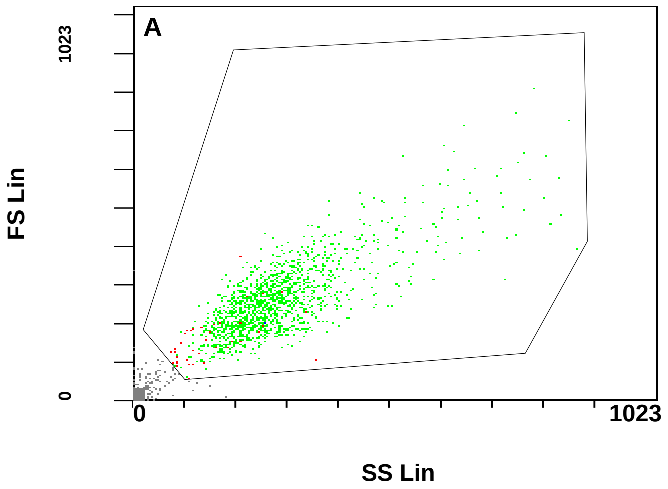

[A] FL3 Lin/FS Lin - ADC

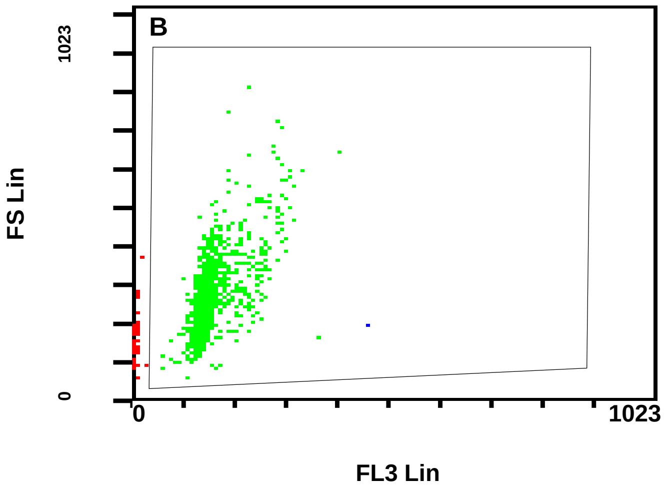

[A AND B] FL3 Lin - ADC

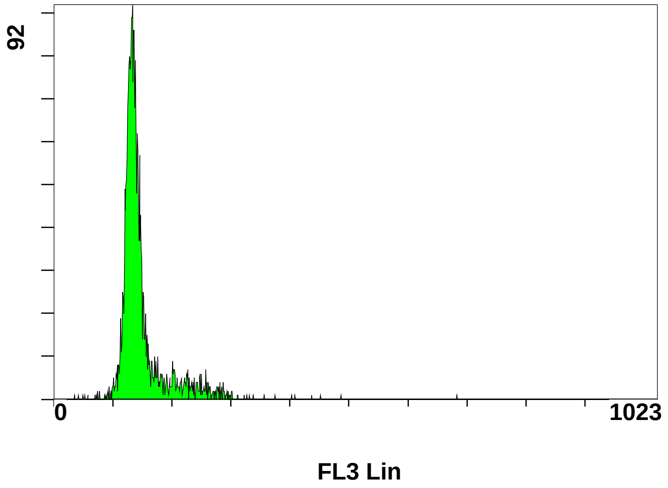

Statistical Analysis

PROGRAM INFORMATION

File:- 10_WJT 00012651 2013-11-20.LMD					
Gate:- A [A]					
Compensation:- Advanced					
Filename:- 10_WJT 00012651 2013-11-20.LMD					
Mean Calculation Method:-LOG-LOG					
Region	Number	%Total	%Gated	X-Mean	Y-Mean
ALL	2866	47.15	100.00	143	263
B	2776	45.67	96.86	148	266

File:- 10_WJT 00012651 2013-11-20.LMD					
Gate:- B [A AND B]					
Compensation:- Advanced					
Filename:- 10_WJT 00012651 2013-11-20.LMD					
Mean Calculation Method:-LOG-LOG					
Region	Number	%Total	%Gated	X-Mean	Y-Mean
ALL	2776	45.67	100.00	148	###

File:- 10_WJT 00012651 2013-11-20.LMD					
Gate:- Ungated					
Compensation:- Advanced					
Filename:- 10_WJT 00012651 2013-11-20.LMD					
Mean Calculation Method:-LOG-LOG					
Region	Number	%Total	%Gated	X-Mean	Y-Mean
ALL	6079	100.00	100.00	139	133
A	2866	47.15	47.15	281	263
