# Supplementary material for: Effects of deuterium oxide on cell growth and vesicle speed in RBL-2H3 cells
Source: PeerJ. 2014 Sep 2;2:e553. doi: 10.7717/peerj.553 (PMC4157235; doi:10.7717/peerj.553)
Supplement: Supplemental Information 4 — LMD files are the Flow Cytometry raw data for Fig. 2.PDF files are overview pages showing analyzed data with the applied gatesFile name description:dayoneH2O: one-day old culture with 0 mol/L deuterium oxide dayfourH2O: four-day old culture with 0 mol/L deuterium oxide dayoneD2O: one-day old culture with 15 mol/L deuterium oxide dayfourH2O: four-day old culture with 15 mol/L deuterium oxide. [file peerj-02-553-s004.zip › Flow Cytometry Data/day_one_D2O.pdf]

Playback Institution:
Protocol :rblcountpianal.PRO
New Protocol
Analysis Date: 13-Nov-2013, 17:05:39
Settings File: rlbcountpifix.PRO, 13-Nov-2013, 16:59:02
Listmode File: 10_WJT 00012631 2013-11-13.LMD

Run Date: 13-Nov-13, 16:59:37
Sample ID: Well 10
User ID: WJT
Acquisition Time/Events: 107.2s / 10000

[Ungated] SS Lin/FS Lin - ADC

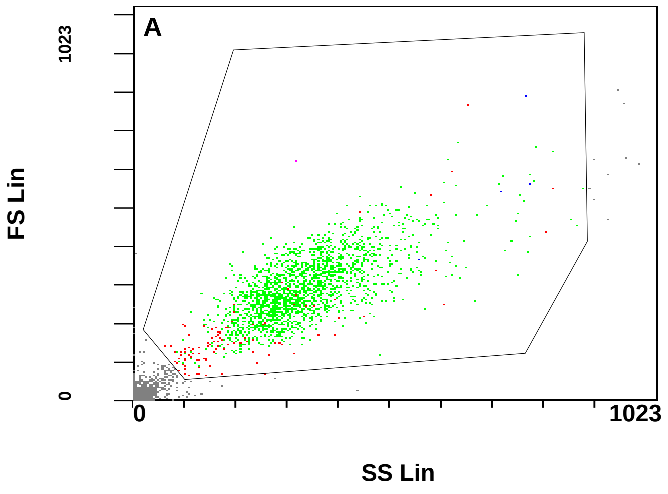

[A] FL3 Lin/FS Lin - ADC

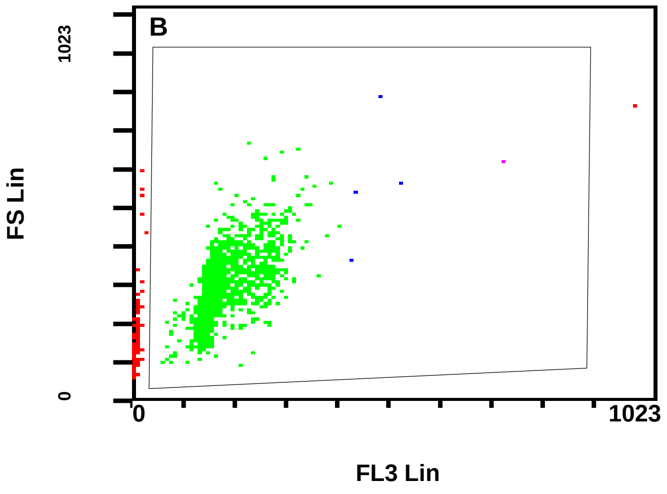

[A AND B] FL3 Lin - ADC

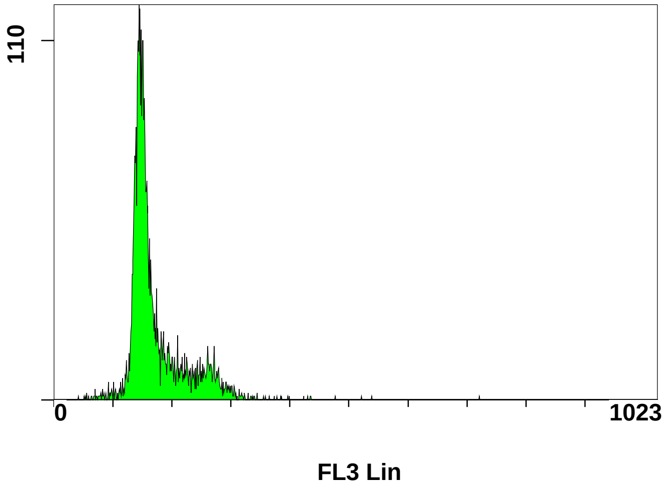

Statistical Analysis

PROGRAM INFORMATION

File:- 10_WJT 00012631 2013-11-13.LMD
Gate:- A [A]
Compensation:- Advanced
Filename:- 10_WJT 00012631 2013-11-13.LMD
Mean Calculation Method:-LOG-LOG

Region	Number	%Total	%Gated	X-Mean	Y-Mean
ALL	4012	40.12	100.00	163	289
B	3800	38.00	94.72	172	295

File:- 10_WJT 00012631 2013-11-13.LMD
Gate:- B [A AND B]
Compensation:- Advanced
Filename:- 10_WJT 00012631 2013-11-13.LMD
Mean Calculation Method:-LOG-LOG

Region	Number	%Total	%Gated	X-Mean	Y-Mean
ALL	3800	38.00	100.00	172	###

File:- 10_WJT 00012631 2013-11-13.LMD
Gate:- Ungated
Compensation:- Advanced
Filename:- 10_WJT 00012631 2013-11-13.LMD
Mean Calculation Method:-LOG-LOG

Region	Number	%Total	%Gated	X-Mean	Y-Mean
ALL	10000	100.00	100.00	142	128
A	4012	40.12	40.12	323	289
